# Acute hypoalgesic, neurophysiological and perceptual responses to low‐load blood flow restriction exercise and high‐load resistance exercise

**DOI:** 10.1113/EP091705

**Published:** 2024-04-05

**Authors:** Ryan Norbury, Ian Grant, Alex Woodhead, Luke Hughes, Jamie Tallent, Stephen D. Patterson

**Affiliations:** ^1^ Faculty of Sport, Technology and Health Sciences St Mary's University Twickenham UK; ^2^ Department of Sport, Exercise and Rehabilitation Northumbria University Newcastle‐Upon Tyne UK; ^3^ School of Sport, Rehabilitation and Exercise Sciences University of Essex Colchester UK; ^4^ Monash Exercise Neuroplasticity Research Unit, Department of Physiotherapy, School of Primary and Allied Health Care, Faculty of Medicine, Nursing and Health Science Monash University Melbourne VA Australia

**Keywords:** blood flow restriction exercise, pain, transcranial magnetic stimulation

## Abstract

This study compared the acute hypoalgesic and neurophysiological responses to low‐load resistance exercise with and without blood flow restriction (BFR), and free‐flow, high‐load exercise. Participants performed four experimental conditions where they completed baseline measures of pain pressure threshold (PPT), maximum voluntary force (MVF) with peripheral nerve stimulation to determine central and peripheral fatigue. Corticospinal excitability (CSE), corticospinal inhibition and short interval intracortical inhibition (SICI) were estimated with transcranial magnetic stimulation. Participants then performed low‐load leg press exercise at 30% of one‐repetition maximum (LL); low‐load leg press with BFR at 40% (BFR_40_) or 80% (BFR_80_) of limb occlusion pressure; or high‐load leg press of four sets of 10 repetitions at 70% one‐repetition maximum (HL). Measurements were repeated at 5, 45 min and 24 h post‐exercise. There were no differences in CSE or SICI between conditions (all *P* > 0.05); however, corticospinal inhibition was reduced to a greater extent (11%–14%) in all low‐load conditions compared to HL (*P *< 0.005). PPTs were 12%–16% greater at 5 min post‐exercise in BFR_40_, BFR_80_ and HL compared to LL (*P *≤ 0.016). Neuromuscular fatigue displayed no clear difference in the magnitude or time course between conditions (all *P *> 0.05). In summary, low‐load BFR resistance exercise does not induce different acute neurophysiological responses to low‐load, free‐flow exercise but it does promote a greater degree of hypoalgesia and reduces corticospinal inhibition more than high‐load exercise, making it a useful rehabilitation tool. The changes in neurophysiology following exercise were not related to changes in PPT.

## Abstract (highlights)

- **What is the central question of this study?**
  Does low‐load, blood flow restricted, resistance exercise promote greater hypoalgesic and neurophysiological changes in the 24 h period post‐exercise, in comparison to high‐load resistance exercise?
- **What is the main finding and its importance?**
  Blood flow restriction exercise augments hypoalgesia compared to free‐flow, low‐load exercise and induces similar hypoalgesia to high‐load exercise, but does not result in any different alterations to corticospinal excitability or inhibition. However, corticospinal inhibition was reduced to a greater extent with low‐load exercise, compared to high‐load exercise. Therefore, low‐load BFR resistance exercise is a suitable method for exercise rehabilitation.

## 1 INTRODUCTION

Performing a bout of exercise can acutely reduce pain sensitivity (Vaegter & Jones, 2020), known as exercise‐induced hypoalgesia (EIH) (Rice et al., 2019), which is typically observed as a transient increase in pressure pain threshold (PPT). In order to produce a hypoalgesia, exercise needs to be of a sufficient intensity and/or duration, pertinent to the exercise modality and the individual (Koltyn, 2002; Vaegter & Jones, 2020). However, the acute exercise variables which are required to optimise the EIH response are not well established, particularly for dynamic resistance exercise (Wewege & Jones, 2021). This is crucial, as an improved understanding of EIH could further the development of resistance exercise as a strategy to reduce pain perception in healthy and clinical populations (Sluka et al., 2018).

Blood flow restriction (BFR) resistance exercise involves performing low‐intensity exercise (<50% one‐repetition maximum) with an inflated tourniquet placed proximally around the limb to restrict arterial inflow and venous return (Patterson et al., 2019). Tourniquets can be inflated to a value relative to the limb occlusion pressure (LOP), defined as the lowest pressure which fully restricts arterial inflow. This method, as opposed to absolute pressures, standardises the degree of arterial occlusion between participants (Evin et al., 2021) and allows for more accurate comparison between studies. Furthermore, LOP is widely recommended for the prescription of BFR pressures for resistance exercise (Patterson et al., 2019; Scott et al., 2023). Therefore, the addition of LOP‐based BFR during low intensity resistance exercise can induce physiological and perceptual responses (i.e., pain and effort) equivalent to or beyond that of traditional high intensity exercise (Patterson et al., 2019; Rossow et al., 2012; Wei et al., 2021). It was recently demonstrated in healthy individuals that BFR resistance exercise can induce a greater degree of EIH (i.e., a greater increase in PPT) than high‐load resistance exercise, but only when high occlusion pressures are used (80% LOP) (Hughes & Patterson, 2020). Furthermore, BFR exercise can reduce patellofemoral pain (Giles et al., 2017; Korakakis et al., 2018), along with improving pain symptoms in patients recovering from anterior cruciate ligament reconstruction (Hughes et al., 2019a, 2019b). Taken together, BFR exercise appears to be a promising method to induce hypoalgesia but has received relatively little attention until recently.

The mechanism(s) which cause EIH from BFR exercise appear to be both central and peripheral in origin (Hughes & Patterson, 2020, 2019). Peripheral mechanisms are, in part, due to elevated levels of endorphins, which bind to opioid receptors in the peripheral nervous system to cause analgesia, potentially caused by the metabolic stress and earlier onset of fatigue, consequently stimulating group III/IV afferents (Song et al., 2021a). Central mechanisms of EIH are of interest as increases in PPT can occur in non‐exercised body parts (Vaegter & Jones, 2020), which could have significant clinical and rehabilitative implications for pain‐related conditions. In particular, EIH may in part be due to γ‐aminobutyric acid type a and b receptor (GABA_a/b_) activity within spinal and supraspinal areas, which is thought to modulate nociceptive transmission (Lau & Vaughan, 2014; Malcangio, 2018). Furthermore, activation of the motor cortex to recruit high threshold motor units during BFR exercise may also play a role in EIH (Senapati et al., 2005; Song et al., 2021a). Single and paired pulse transcranial magnetic stimulation (TMS) makes it possible to non‐invasively investigate the neurophysiology of the corticospinal pathway, which can elucidate the central mechanisms of EIH. Specifically, GABA_b_ activity is reflected by the duration of the TMS silent period (Škarabot et al., 2019; Ziemann et al., 2015), and acute pain has been shown to increase the duration of the TMS silent period duration (de Almeida Azevedo et al., 2022; Norbury et al., 2022). Decreases in short interval intracortical inhibition (SICI), which is reflective of GABA_a_ activity (Ziemann et al., 2015), have been observed in those with patellar tendinopathy after exercise‐induced hypoalgesia (Rio et al., 2015). Corticospinal excitability (CSE), indicated by the motor evoked potential (MEP) amplitude is also associated with more efficient conditioned pain modulation, and temporal summation (Granovsky et al., 2019). As a result of these observations, indices derived from TMS (e.g., silent period, SICI and MEP_amp_) can be used to explore the central mechanisms of EIH.

Currently, the centrally mediated mechanisms which are responsible for BFR exercise decreasing pain sensitivity are not fully known (Song et al., 2021a). Endogenous opioids have been implicated, but this has not fully explained the systemic hypoalgesic response to BFR exercise (Hughes & Patterson, 2020). Furthermore, the acute, corticospinal changes to BFR resistance exercise within the quadriceps muscle have not been investigated (Brandner et al., 2015). This is of particular interest because BFR quadriceps exercise is frequently used within clinical (Ladlow et al., 2018; Tennent et al., 2017) and performance settings (Clark et al., 2011). Therefore, the aim of the present study was to investigate the acute hypoalgesic, neurophysiological and perceptual responses to low‐load BFR exercise at low and high arterial occlusion pressures in comparison to free‐flow low‐load exercise and free‐flow high‐load exercise. We hypothesised that BFR exercise would induce a greater increase in PPT compared to non‐BFR and high‐load exercise, and this will be accompanied by a greater increase in CSE and reduced corticospinal inhibition. However, we expect that BFR exercise will be more acutely fatiguing and more painful than free‐flow exercise.

## 2 METHODS

### 2.1 Participants

Twelve male participants (mean ± SD age 29 ± 6 years; height 1.80 ± 0.06 m; body mass 85.4 ± 13.6 kg; unilateral leg press one‐repetition maximum 184 ± 40 kg) volunteered to take part in the study after providing written informed consent. All participants were healthy, pain free, and did not display any contraindications to TMS, as assessed by the questionnaire from Rossi et al. (2011), nor did participants exhibit contraindications to BFR exercise as indicated by a physical activity readiness questionnaire and a study‐specific questionnaire to screen for cardiovascular conditions (e.g., deep vein thrombosis). All participants were recreationally active and familiar with performing resistance exercise, but not all participants performed regular resistance training. Prior to recruitment, this study received ethical approval from the university ethical committee (ref: SMU_ETHICS_2021‐22_269) and was conducted in accordance with the declaration of Helsinki, but without being registered.

### 2.2 Sample size justification

The sample size required was determined a priori in G*Power 3.1.9.7 (Faul et al., 2007). An effect size from a previous study was calculated (Brandner et al., 2015), which compared MEP amplitude normalised to M‐wave amplitude (indicative of CSE) between low‐load BFR and low‐load, free‐flow exercise at 5 min post‐exercise (Cohen's *f *= 0.87). To detect an effect size of this magnitude at a power of 0.8, using an α‐level of 0.05, with four conditions in a repeated measures analysis of variance, correlation among repeated measures 0.5 and non‐sphericity correction of 1, a sample of *n *= 8 was required. To partially account for the overestimation of effect sizes present in published literature, due to publication bias (Lakens, 2022), and because corticospinal projections may be less in the lower compared to upper limb (Brouwer & Ashby, 1990), a more conservative Cohen's *f* of 0.4 was used which coincides with the threshold for ‘large effect’ (Cohen, 2013). This ‘large effect’ aligns with our assumption that BFR will induce large effects on our measures of interest. Therefore, with the same parameters, an *n *= 12 was required and subsequently recruited. This sample size exceeds that of Brandner et al. (2015) of *n *= 10. Our use of a linear mixed effects model for statistical analysis with the inclusion of baseline covariates increased statistical power beyond the calculations above.

### 2.3 Experimental procedures

**FIGURE 1:**
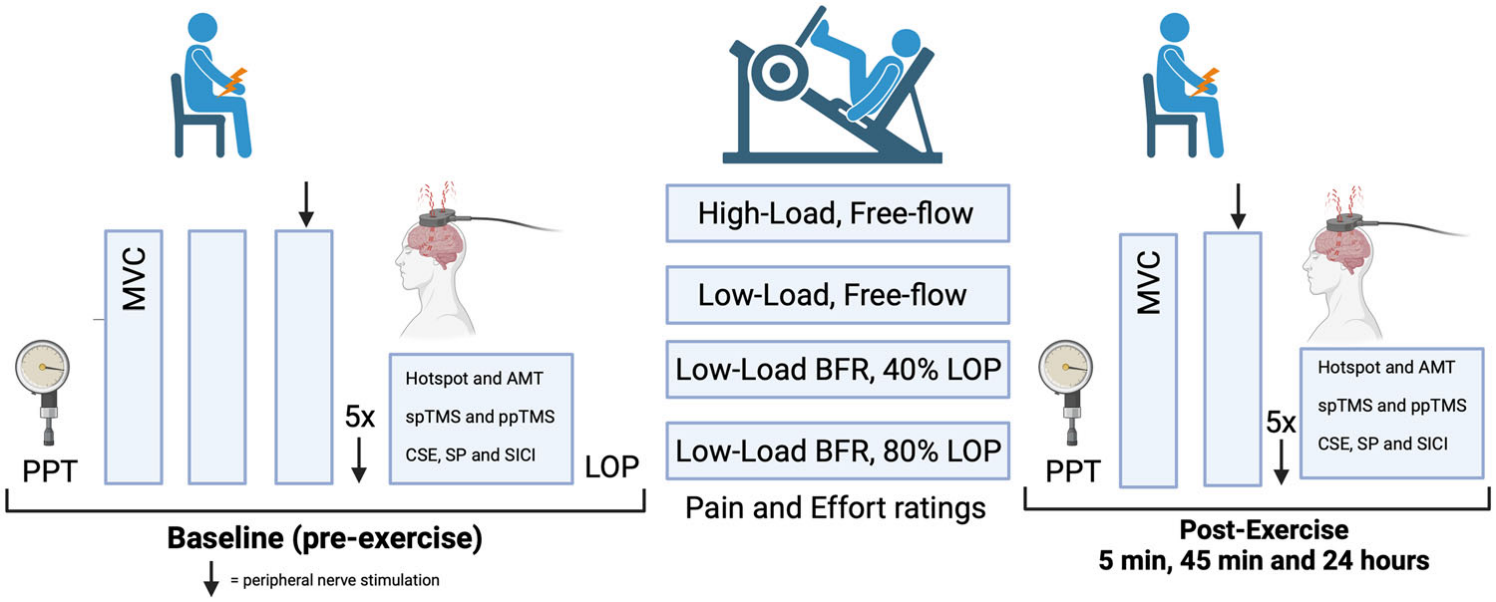
Schematic representation of the experimental procedures. AMT, active motor threshold; BFR, blood flow restriction exercise; CSE, corticospinal excitability; LOP, limb occlusion pressure; MVC, maximum voluntary contractions; PPT, pain pressure threshold; ppTMS, paired pulse transcranial magnetic stimulation; SICI, short interval intracortical inhibition; spTMS, single pulse transcranial magnetic stimulation; SP, TMS silent period.

In a randomised, crossover, single blinded (experimenter) repeated measures design, participants visited the laboratory on nine separate occasions at a similar time of day (±2 h) for one familiarisation and eight experimental visits across four different conditions. Participants attended the laboratory in a rested state (no strenuous lower body exercise 48 h before), and did not consume caffeine (8 h), alcohol (24 h), or analgesics (24 h) before testing. A schematic representation of the experimental visits is depicted in Figure 1.

#### 2.3.1 Visit 1. Baseline measures and familiarisation

Participants completed an initial visit which included completion of health screening forms and collection of anthropometric data. Participants were then familiarised with all experimental procedures and measures. Unilateral leg‐press one‐repetition maximum (1RM) of the right leg was conducted under supervision of a qualified personal trainer, in line with American College of Sports Medicine (2013) guidelines. Briefly, participants performed five repetitions from full extension to 90‐degree knee flexion at increments of 25 kg until the weight was perceived to be ‘hard’. Increments of 10–20 kg for three repetitions were performed, before finally increments of 2.5–10 kg for one repetition until the maximum weight the participants could lift was established. Once 1RM was established, participants were familiarised with BFR exercise and metronome‐paced leg press exercise by performing the BFR protocol at 80% of LOP. This condition was selected to ensure participants were willing to tolerate the pain and effort induced by the high pressure BFR exercise, and to provide familiarisation for ratings of pain and effort.

#### 2.3.2 Visits 2–9. Experimental conditions

Initially, participants arrived at the laboratory and were seated in a custom‐built isometric chair. Measurements of PPT were taken on the right and left quadriceps muscle, the right bicep and the left trapezius. Peripheral nerve stimulation was then performed where participant's optimal stimulation site and intensity were determined. Participants then underwent a baseline measure of neuromuscular function. Firstly, participants performed three warm‐up contractions at 50%, 75% and 90% of their perceived maximum effort (5 s contraction, 30 s rest) before performing three MVCs interspersed with 2 min of rest with one superimposed and five resting potentiated peripheral nerve stimulations after the final MVC (see section 2.9, ‘Peripheral nerve stimulation’ below for details). Transcranial magnetic stimulation was then performed where the optimal stimulation site was initially found before the active motor threshold, CSE, corticospinal inhibition and short interval intracortical inhibition were measured (see section 2.8, ‘Transcranial magnetic stimulation’ below for details).

Upon completion of baseline measures, participants moved into a separate room to the unilateral leg press machine and had their LOP measured after lying supine for 5 min. A warm‐up set on the unilateral leg press was then completed by performing one set of 15 repetitions at 15% of 1RM. After 2 min of rest, participants then completed one of the leg press exercise protocols, which was randomised for each participant using a random number generator in Microsoft Excel. This was one of high‐load, free‐flow exercise (HL), low‐load, free‐flow exercise (LL), low‐load BFR exercise at 40% of LOP (BFR_40_) or low‐load BFR exercise at 80% LOP (BFR_80_) (see section 2.5, ‘Leg press exercise’ below for more detail). After completion of the leg press exercise (approximately 6 min duration), participants were seated back in the isometric chair where measures were conducted for the first post‐exercise time point (5 min). These included measures of PPT, two MVCs with peripheral nerve stimulation on and after the second MVC, along with TMS measures (Figure 1). This post‐exercise protocol was performed again at 45 min 24 h, except at the 24‐h time point, the optimal peripheral nerve stimulation site and intensity was re‐established along with the TMS hotspot.

### 2.4 Blood flow restriction

An automated personalised tourniquet system (Delfi Medical Inc., Vancouver, BC, Canada) was used to restrict blood flow to the right leg. This BFR device automatically measures LOP, defined as the minimum pressure required for full arterial occlusion of the limb by monitoring arterial pulsations in response to increments of tourniquet pressure, and exhibits excellent agreement (mean difference 95% confidence intervals: −3 to +3 mmHg) with the Doppler ultrasound technique (Masri et al., 2016). This system is a dual‐purpose (i.e., measures LOP and restricts blood flow) contour cuff (11.5 cm width and 86 cm length) connected by tubing to a pneumatic tourniquet device that automatically regulates pressure in response to transient pressure spikes induced by muscular contractions during exercise (Hughes et al., 2024; Lai et al., 2023). The cuff was applied to the most proximal portion of the limb. LOP was measured in the BFR_40_ and BFR_80_ condition in the supine position after 5 min of quiet rest (Hughes et al., 2018). The tourniquet was inflated 3 s before exercise to a pressure of 40% LOP in BFR_40_ and 80% LOP in BFR_80_ to allow for the desired pressure to be reached at the onset of exercise. These percentages of LOP are regarded as low and high‐pressures for use of lower body resistance exercise (Patterson et al., 2019). Once the exercise protocol was finished, the cuff was deflated, and therefore the cuff remained inflated during the interest rest periods.

### 2.5 Leg press exercise

Participants performed four sets of resistance exercise with their right leg on a unilateral leg press machine (Pullum ISO Incline 45 Leg Press, Pullum Sports, Leighton Buzzard, UK). The exercise protocol for LL, BFR_40_ and BFR_80_ was 30, 15, 15 and 15 goal repetitions from sets 1–4, respectively, at 30% 1RM with 30 s rest between sets. Within HL, the protocol was four sets of 10 goal repetitions at 70% 1RM with 53 s of rest between sets to be of a similar duration to the low‐load protocols (Hughes & Patterson, 2020). Each repetition was performed from full extension to a 90‐degree knee flexion range of motion at a cadence of 1.5 s concentric, 0 s isometric and 1.5 s eccentric to which participants were guided with a metronome.

### 2.6 Pain pressure threshold

Initially, within‐session reliability of PPTs was determined during the familiarisation to demonstrate stability of the measurements on acute, repeated recordings for the primary investigator. Participants were seated on the isometric chair with their feet off the ground, torso upright and arms relaxed in the lap supinated. Two measurements were taken on the right (exercised) and left (non‐exercised) quadricep (marked at 20 cm proximal to the base of the patella) and the right biceps brachi (10 cm proximal to the cubital fossa) as well as the left upper trapezius muscle (10 cm from the acromion in direct line with the neck) using a handheld digital algometer (FDX 50, Wagner Instruments, Greenwich, CT, USA). These measurements provide a comprehensive assessment of local and systemic hypoalgesia, including ipsilateral, contralateral and non‐homologous muscle groups, which allow for greater comparisons between studies, particularly to Hughes and Patterson (2020) who used the same sites. Pressure was applied manually with a 1 cm^2^‐diameter stimulation site at an ascending rate of approximately 1 kg force per second. Participants were instructed to verbally indicate when they first perceived the pressure stimulus as painful. An interval of 30 s was given between measurement sites and if the two measurements were >10% apart, then a third measurement was taken and the mean of the closest two values was used. Within‐sessions reliability of the PPTs can be seen in Table 1 below.

**TABLE 1:** Within‐session reliability of PPT measurements at each site.

	Within‐day reliability		
	CV (%)	SEM (kg)	MDC (kg)
Right quadricep	4.1 (2.3–5.9)	0.25 (0.13–0.36)	0.69 (0.38–1.01)
Left quadricep	7.8 (5.0–10.6)	0.35 (0.26–0.45)	0.98 (0.70–1.25)
Right bicep	8.6 (5.1–12.2)	0.17 (0.09–0.26)	0.46 (0.26–0.71)
Left trapezius	7.2 (4.7–9.7)	0.22 (0.13–0.30)	0.60 (0.35–0.84)

*Note*: Data presented as mean and 95% confidence interval. CV% calculated as mean values divided by standard deviation multiplied by 100. SEM calculated as standard deviation of measures divided by the square root of the number of measures. MDC calculated as SEM multiplied by 1.96 multiplied by the square root of two. Abbreviations: CV, coefficient of variation; MDC, minimum detectable change; SEM, standard error of measurement.

### 2.7 Surface electromyography

Bipolar surface electromyography (sEMG) was recorded for the right rectus femoris (RF) with 22 × 22 mm (interelectrode distance = 22 mm) surface electrodes (Ambu WhiteSensor 40554; Ambu Ltd, Ballerup, Denmark). Initially, the site was shaved, abraded and cleaned before the electrodes were placed 50% of the distance between the anterior spina iliaca superior to the superior part of the patella (Hermens et al., 1999). The location was slightly adjusted to improve the signal‐to‐noise ratio when necessary. This site was marked with indelible ink to ensure reproducible placement location. Surface electromyography data were recorded at a frequency of 2.5 kHz (CED Micro1401, Cambridge Electronic Design, Cambridge, UK) amplified (D440‐2; Digitimer, Welwyn Garden City, UK; gain = 1000) and band pass filtered (10–1000 Hz).

### 2.8 Transcranial magnetic stimulation

Single‐pulse and paired‐pulse TMS was delivered using two magnetic stimulators (Magstim Bistim, The Magstim Company Ltd, Whitland, UK) with a double cone coil which was placed over the left side of the motor cortex to induce a posterior–anterior current flow for generating MEPs in the right RF. Initially, participants wore a Lycra swimming cap where the vertex was marked, which was identified as the tragus and nasal‐inion intersect. Subsequently, the TMS coil centre was placed at this position and systematic deviations were made 2 cm posterior and 2 cm anterior of the vertex, in 1 cm alterations. The point which evoked the greatest RF MEP peak‐to‐peak amplitude (mV) was identified on this axis, then 1 cm movements were performed from the midline to 2 cm laterally and the point with the greatest MEP peak‐to‐peak amplitude was used, and this point was defined as the hotspot. These initial stimulations were delivered at 40%–50% of maximum stimulator output (MSO) during an isometric contraction of the knee extensors at an intensity of 10% of maximum voluntary force (MVF). The location of the hotspot was marked onto the swimming cap for all subsequent stimulations.

The active motor threshold (AMT) during a 10% MVF isometric knee extensor contraction was determined by delivering five stimuli, beginning at 35% MSO and progressing in 5% increments or decrements until the lowest intensity was found which evoked a visible RF MEP of >0.2 mV peak to peak amplitude in at least three out of five stimulations (Ansdell et al., 2020). The stimulation intensity was then altered by steps of 1% until this criterion was achieved to the exact percentage.

Single pulse TMS was used to produce MEPs at an intensity of 130% and 150% of AMT in the rectus femoris during intermittent 10% MVF isometric knee extensor contractions (3 s contracting, 3 s relaxation). A total of 10 stimuli were delivered once the force had plateaued at the target. Paired pulse TMS was also delivered to induce SICI and stimuli were delivered using the same method as single pulse except a conditioning stimulus was delivered at 70% AMT followed by the test stimulus at 130% AMT (inter‐stimulus interval = 3 ms). These parameters have been shown to be suitable to induce SICI (Brownstein et al., 2018).

### 2.9 Peripheral nerve stimulation

Square wave electrical stimuli were delivered with a constant current electrical stimulator (DS7AH, Digitimer; maximum voltage = 400 V; pulse duration = 200 μs). The anode was a 32 × 32 mm circular self‐adhesive neurostimulation electrode (Axelgaard Manufacturing, Lystrup, Denmark), which was attached to the gluteal fold. The cathode was another 32 × 32 mm self‐adhesive electrode, which was placed within the femoral triangle (approximately 2.5 cm medial and inferior to the anterior superior iliac spine), and the position of the cathode was adjusted in 1 cm alterations to a position which evoked a visible twitch response at 100 mA. Once this site was found, stepwise increments of electrical stimuli of 20 mA beginning at 80–100 mA were performed until there was a plateau in the twitch force and M‐wave peak‐to‐peak amplitude (*M*_max_) was observed. The stimulation amplitude was further increased by 20% and checked to ensure *M*_max_ was obtained (Osborne et al., 2023). This procedure was repeated at the post‐24‐h time point.

### 2.10 Perceptual measures

#### 2.10.1 Pain intensity

The intensity of perceived pain was recorded with the 11‐point (0–10) Cook pain scale (Cook et al., 1998) with 0 as ‘no pain at all’ and 10 at ‘extremely intense pain (almost unbearable)’. Participants were asked to rate pain intensity perceived at the end of each set of exercises and were explicitly instructed to anchor their upper pain rating to ‘the worst exercise‐induced pain you have ever felt’. This was to avoid participants from using a pain anchor from other painful experiences (e.g., muscle injury) or from anchoring their pain to an imagined theoretical maximum intensity.

#### 2.10.2 Rating of perceived effort

The perception of effort was recorded on the Borg 6–20 scale (Borg, 1998) at the end of each set. Participants were instructed to select their rating based upon the ‘effort to drive the limb’ to perform the final repetition of the set, and to anchor their upper rating of 20 ‘maximal effort’ with the effort to drive the limb required during their 1RM in the familiarisation session (Lopes et al., 2022; Pageaux, 2016). Importantly, participants were also instructed to not include feelings of pain, fatigue or discomfort within the rating of perceived effort (RPE) measurement.

### 2.11 Data analysis

PPT was taken as the mean of the two recordings. The mean of the MVF and potentiated twitch force (*Q*_tw_) was taken as the peak‐to‐peak instantaneous force achieved during an MVC and twitch, respectively. Voluntary activation was calculated as (Strojnik & Komi, 1998):

$$\begin{array}{ccc} 100 & - & \mathrm{superimposed}\;\mathrm{twitch}\;\mathrm{force}\;\left(n\right)\\ & & \frac{\left(\frac{\mathrm{force}\;\mathrm{before}\;\mathrm{twitch}\;\left(n\right)}{\mathrm{peak}\;\mathrm{force}\;\left(n\right)}\right)}{\mathrm{resting}\;\mathrm{potentiated}\;\mathrm{twitch}\;\mathrm{force}\;\left(n\right)}\;\times 100 \end{array}$$

To reflect CSE, an average of the peak‐to‐peak amplitude of the MEPs normalised to the mean of the peak‐to‐peak amplitude of the *M*_max_ × 100 (MEP·*M*_max_) was calculated. Corticospinal inhibition was reflected with the duration of the TMS silent period, which was visually inspected from the point of the stimulus artefact until resumption of voluntary sEMG activity. SICI was represented as:

$$\left(1-\;\frac{\mathrm{mean}\;\mathrm{of}\;\mathrm{conditioned}\;\mathrm{MEPs}\;\left(\mathrm{mV}\right)}{\mathrm{mean}\;\mathrm{of}\;\mathrm{unconditioned}\;\mathrm{MEPs}\;\left(\mathrm{mV}\right)}\right)\times 100$$

whereby a lower number reflected less inhibition (Lackmy & Marchand‐Pauvert, 2010). All data were analysed by the same investigator (R.N.) who was blinded to the experimental condition the participants underwent. Blinding was achieved by having a separate investigator conduct the leg press exercise with the participant in a separate room from where the post‐exercise measures were performed. Data files were subsequently coded to a number corresponding to the trial the participant had completed for that session and was revealed after data analysis was completed.

### 2.12 Statistical analysis

All data were statistically analysed in JAMOVI 2.3.13 (The Jamovi Project, 2019). Initially, the standardised residuals of the dependent variables were checked for normality with the Kolmogorov–Smirnov test, Q‐Q plots and histograms. If the assumption of normality was not reasonably satisfied, the raw data values were log_10_ transformed (SP130 and SP150; data presented without transformation for ease of interpretation). Corticospinal (MEP·*M*_max_, Silent Period, SICI) neuromuscular (MVF, VA, *Q*_tw_, *M*_max_), PPT and perceptual (Pain and Effort) was analysed with a repeated measures linear mixed model using the ‘gamlj’ package in JAMOVI with restricted maximum likelihood. Time (5 min, 45 min and 24 h) or set (1, 2, 3 and 4 for perceptual measures) and condition (LL, HL, BFR_40_ and BFR_80_) with condition × time/set interactions were included as fixed effects, with individual participant intercepts included as a random effect. To account for between‐session variability in dependent variables, the baseline value was included as a centred covariate. Also, to account for differences in leg‐press exercise volume between conditions, the achieved volume in kg (load × repetitions completed) was also included as a centred covariate in the model, except for pain and RPE modelling. Upon a statistically significant interaction, simple effect analysis was performed to determine differences at each level of each factor. In the absence of interaction effects, post‐hoc tests were performed for statistically significant main effects of condition and were Holm–Bonferroni corrected for multiplicity (Holm, 1979). An intraclass correlation coefficient (ICC 3,1) was used to assess relative reliability in LOP between conditions, with ICC of >0.5, >0.75 and >0.90 regarded as moderate, good and excellent reliability, respectively (Koo & Li, 2016). Statistical significance was set at *P *< 0.05. Effect sizes were reported as Cohen's *d_z_* and was calculated as the *t*‐statistic divided by the square root of the sample size. Values of 0.2, 0.5 and 0.8 represented thresholds for small, medium and large effects, respectively (Cohen, 2013). Pre to post effect sizes with supplementary 95% confidence intervals were also included to indirectly infer changes over time within each when a condition or interaction, or time effect was present. All data are presented as mean ± SD unless stated otherwise. Individual participant's data for each dependent variable can be viewed within the supplementary material (https://doi.org/10.6084/m9.figshare.24156741).

## 3 RESULTS

Of the 24 BFR trials, one participant experienced a minor injury from the cuff compression during BFR_80_ and they were able to repeat the trial 1 week later. An overview of the exercise variables and BFR pressures can be seen in Table 2. The LOP in BFR_40_ was 184 ± 22 mmHg and 187 ± 24 mmHg in BFR_80_, which demonstrated low intra‐individual variability (CV = 4.1 ± 3.1%) and an ICC of 0.858 [95% CI: 0.596, 0.957] between conditions, indicating moderate to excellent reliability of LOP calculations with a point estimate of ‘very good’ reliability.

**TABLE 2:** Overview of the Leg‐Press exercise performance variables and BFR pressures used.

	BFR pressure (mmHg)	Load (kg)	Set 1	Set 2	Set 3	Set 4	Volume (kg)
LL	—	54 ± 12	30 (0)	15 (0)	15 (0)	30 (0)	4134 ± 899
HL	—	126 ± 28	10 (0)	10 (0)	10 (0)	10 (0)	5065 ± 1031
BFR_40_	74 ± 9	54 ± 12	30 (0)	15 (0)	15 (0)	14 (0)	4066 ± 943
BFR_80_	150 ± 19	54 ± 12	30 (0)	15 (0)	12 (1)	8 (12)	3636 ± 981

*Note*: Data are means ± SD for BFR pressures, and exercise volume completed and medians (interquartile range) repetitions completed for each experimental condition.

Abbreviation: BFR, blood flow restriction.

### 3.1 Exercise‐induced hypoalgesic responses

**FIGURE 2:**
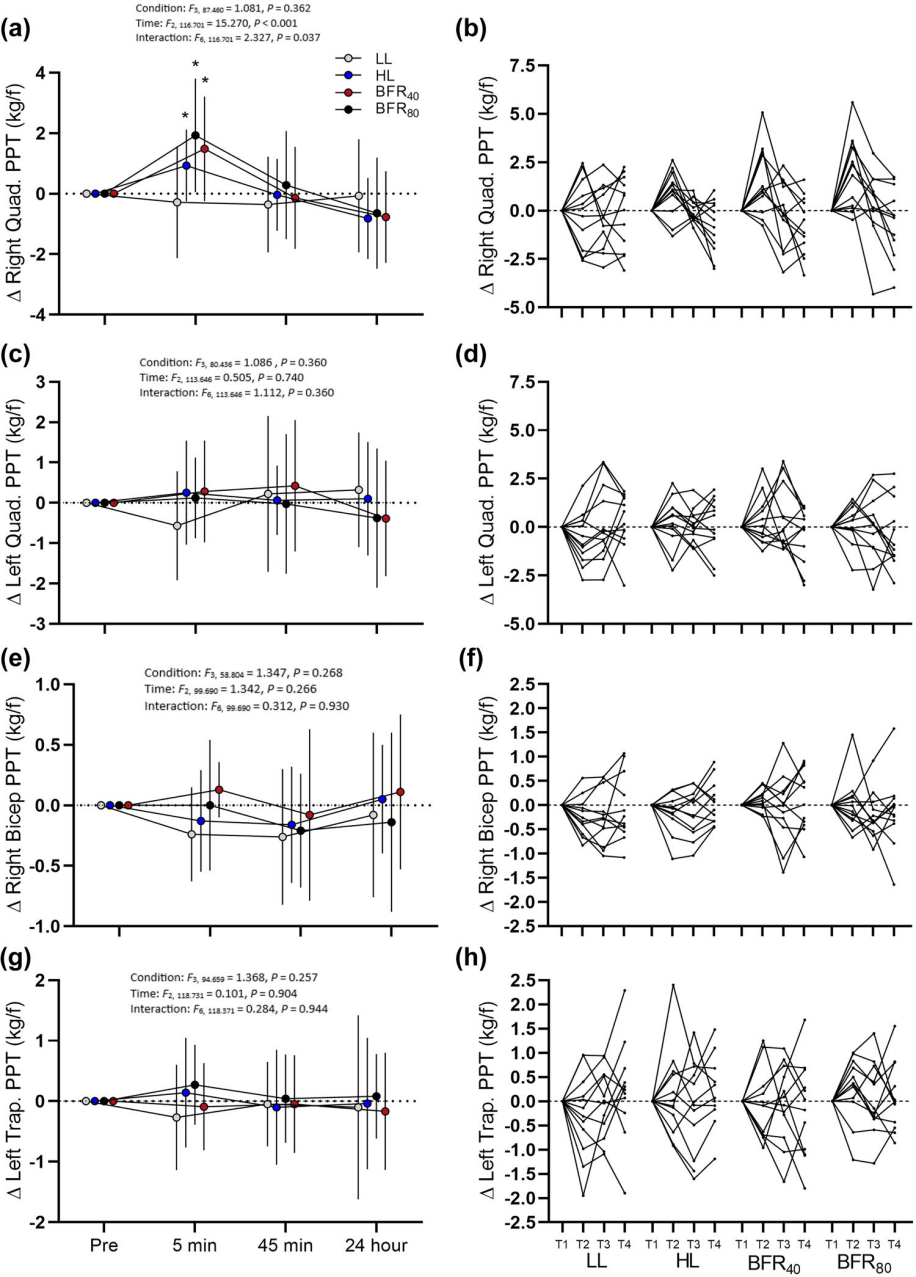
Pain pressure threshold changes in response to each exercise condition. (a) Mean ± SD of right quadricep (exercised leg). (b) Individual changes of right quadricep quadricep PPT. (c) Mean ± SD of left quadricep. (d) Individual changes of left quadricep. (e) Mean ± SD changes of right bicep. (f) Individual changes of right bicep. (g) Mean ± SD of left trapezius. (h) Left trapezius individual changes. T1, T2, T3 and T4 reflect baselines, 5 min, 45 min and 24 h post‐exercise time points, respectively. *Significantly different from LL (interaction effect, *P *< 0.05). kg/f, kg force.

For the right (exercised) quadricep, a condition × time interaction was observed (*F*_6,116.701_ =  2.327, *P* =  0.037). Simple effects analysis revealed that at 5 min post‐exercise, PPT was 14% greater in BFR_40_ compared to LL (mean diff. = 1.67 kg [95% CI: 0.50, 2.83 kg], *t*_117.202_ = 2.834, *P* = 0.005, *d_z_* = 0.82), and was 16% greater in BFR_80_ compared to LL (mean diff. = 1.94 kg [95% CI: 0.75, 3.14 kg], *t*_124.355_ = 3.232, *P* = 0.002, *d_z_* = 0.93). There was no difference in PPTs at 5 min between BFR_40_ and BFR_80_ (mean diff. = −0.25 kg [95% CI: −1.43, 0.94 kg], *t*_122.592_ = −0.409, *P* = 0.645, *d_z_* =  0.12). PPT at 5 min was also 12% greater in HL compared to LL (mean diff. = 1.54 kg [95% CI: 0.29, 2.79 kg], *t*_129.654_  =  2.440, *P* =  0.016, *d_z_* = 0.70), but there was no difference between HL and BFR_40_ or BFR_80_ (mean diff. = −0.26 kg [95% CI: −1.44, 0.91 kg], *t*_111.484_ = −0.449, *P* = 0.654, *d_z_* = 0.13). From baseline to 5 min effect size for PPT in LL was −0.15 [95% CI: −0.72, 0.42], in HL was 0.78 [95% CI: .11, 1.42], in BFR_40_ was 0.86 [95% CI: 0.18, 1.51] and in BFR_80_ was 1.04 [95% CI: 0.3, 1.73]. PPT was not different between any condition at 45 min (*P *≥ 0.307, *d_z_ ≤ *0.30) or 24 h (*P *≥ 0.161, *d_z_ ≤ *0.41). All other PPT assessment sites revealed no changes over time or between conditions (all *P *> 0.05; Figure 2c, e, and g).

### 3.2 Corticospinal responses

#### 3.2.1 MEP·*M*_max_ at 130% AMT

**FIGURE 3:**
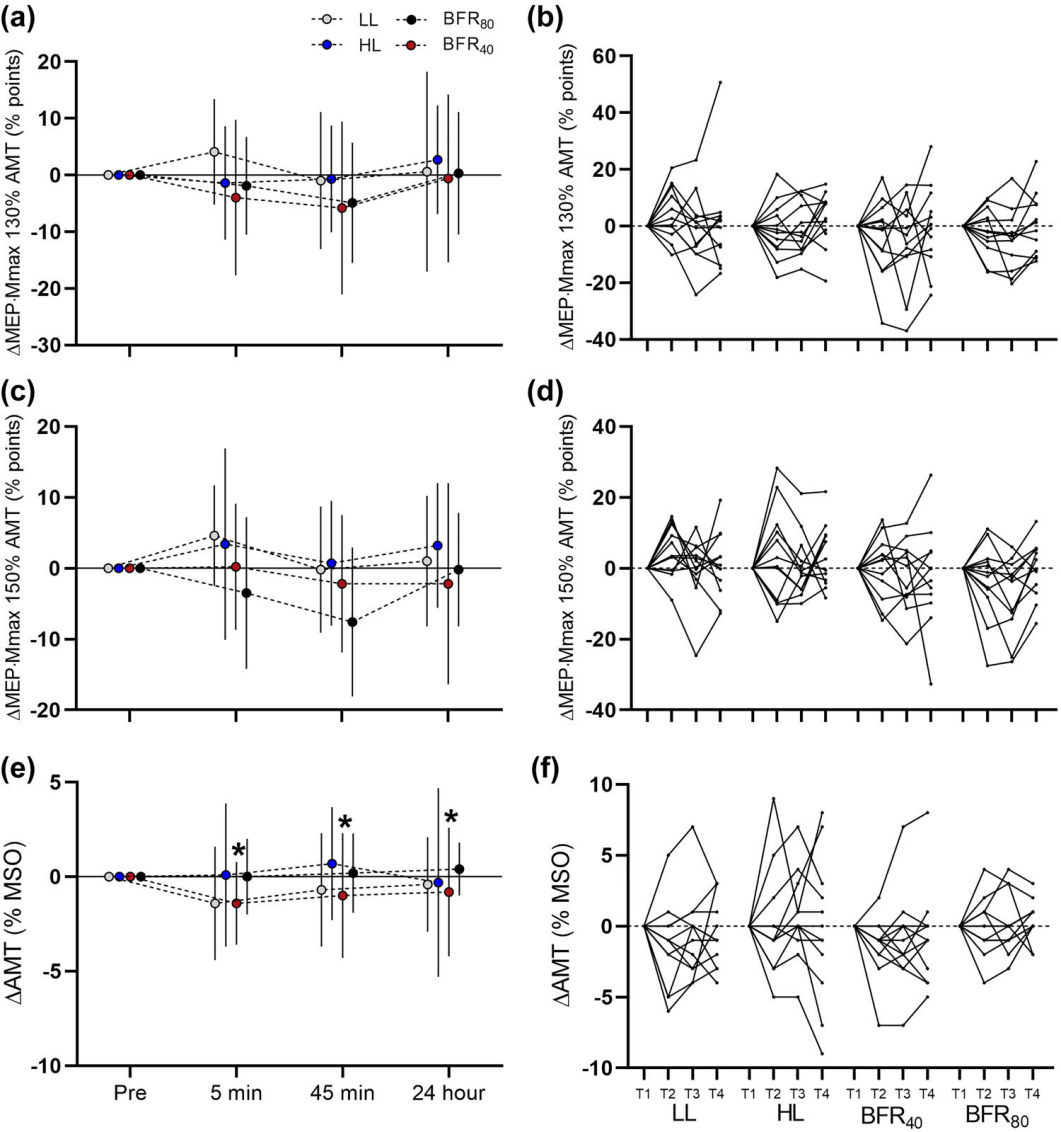
Change of excitability measures derived from TMS values over each time point in each experimental condition. (a) Mean ± SD of MEP·*M*_max_^−1^ ratio (indicative of corticospinal excitability) at 130% of active motor threshold. (b) Individual traces of MEP·*M*_max_^−1^ at 130% active motor threshold. (c) Mean ± SD of MEP·*M*_max_^−1^ ratio at 150% of active motor threshold. (d) Individual traces of MEP·*M*_max_^−1^ at 150% active motor threshold. (e) Mean ± SD active motor threshold. (f) Individual traces of active motor threshold. T1, T2, T3 and T4 denote pre, 5 min, 45 min and 24 h time points, respectively. *Significantly different from HL (condition effect *P *< 0.05).

No condition × time interaction was observed (*F*_6,115.831_ = 0.433, *P* = 0.855). There was also no main effect of condition (*F*_3,107.508_ = 1.760, *P* =  0.161) or main effect of time (*F*_2,115.831_ = 1.856, *P* =  0.161) (Figure 3a).

#### 3.2.2 MEP·*M*_max_ at 150% AMT

Similarly, there was no interaction effect for responses at 150% AMT (*F*_6,117.334_ = 0.596, *P* = 0.733), nor was there a main effect of condition (*F*_3,105.694_ = 1.106, *P* = 0.350) or time (*F*_2,117.334_ = 2.351, *P* =  0.100) (Figure 3c).

#### 3.2.3 Active motor threshold

There was no interaction effect (*F*_6,116.017_ = 0.273, *P* = 0.948) or time effect (*F*_2,116.017_ = 0.638, *P* = 0.530). A condition effect was observed (*F*_3,121.085_ = 3.458, *P* = 0.019), with post‐hoc tests revealing a lower AMT in BFR_40_ (mean = 33% MSO [95% CI: 31, 35% MSO]) compared to HL (mean = 35% MSO [95% CI: 33, 37% MSO]) (*P *= 0.023, *d_z_ *= 0.72). All other comparisons between conditions were not significant (*P *≥ 0.100, *d_z_* ≤ 0.69).

#### 3.2.4 TMS silent period at 130% AMT

No condition × time interaction was observed for the silent period (*F*_6,112.717_ = 0.295, *P* =  0.938). However, there was a main effect of condition (*F*_3,117.082_ = 5.468, *P* =  0.001) and a main effect of time (*F*_2,112.717_ = 9.578, *P* <  0.001). Collapsed across time, the silent period was greater in HL (mean = 151 ms [95% CI: 138, 165 ms]) compared to LL (mean = 136 ms [95% CI: 125, 147 ms], *P *= 0.003, *d_z_* = 1.01), BFR_40_ (mean = 133 ms [95% CI: 123–145 ms], *P *= 0.001, *d_z_* = 1.11) and BFR_80_ (mean = 132 ms [95% CI: 21, 143 ms], *P *= 0.004, *d_z_* = 0.98), with no other differences between conditions (all *P *≥ 0.753, *d_z_* ≤ 0.33). Effect sizes for pre to 5 min were 1.40 [95% CI: 0.57, 2.19] in LL, 0.42 [95% CI: −0.19, 1.00] in HL, 0.83 [95% CI: 0.15, 1.47] in BFR_40_ and 0.73 [95% CI: 0.08, 1.36] in BFR_80_. From pre to 45 min effect sizes were 0.69 [95% CI: 0.04, 1.31] in LL, 0.18 [95% CI: −0.45, 0.68] in HL, 0.39 [95% CI: −0.21, 0.97] in BFR_40_ and 0.80 [95% CI: 0.13, 1.44] in BFR_80_. From pre to 24 h effect sizes in LL were 0.45 [95% CI: −0.16, 1.03], in HL −0.138 [95% CI: −0.70, 0.43], in BFR_40_ 0.24 [95% CI: −0.34, 0.81] and in BFR_80_ 0.49 [95% CI: −0.12, 1.08].

#### 3.2.5 TMS silent period at 150% AMT

No condition × time interaction was observed for the silent period at 150% AMT (*F*_6,119.172_ = 0.475, *P* = 0.826), but there was a main effect of condition (*F*_3,118.714_ = 6.818, *P* < 0.001) and a main effect of time (*F*_2,119.172_ = 9.324, *P*  < 0.001). Post‐hoc revealed that the silent period was greater in HL (mean = 152 ms [95% CI: 142, 163 ms]) compared to BFR_40_ (mean = 147 ms [95% CI: 137, 157 ms], *P *= 0.007, *d_z_* = 0.96). Additionally, the silent period was lower in BFR_40_ compared to BFR_80_ (mean = 157 ms [95% CI: 146, 168 ms], *P *= 0.024, *d_z_* = 0.83). No other differences were observed between condition (*P *≥ 0.105, *d_z_* ≤ 0.65). Effect size from pre to 5 min in LL was 1.00 [95% CI: 0.29, 1.69], in HL was 0.398 [95% CI: −0.20, 0.98], in BFR_40_ was 0.94 [95% CI: 0.24, 1.61] and in BFR_80_ was 0.362 [95% CI: −0.23, 0.94]. From pre to 45 min effect size was 0.52 [95% CI: −0.09, 1.12] in LL, 0.05 [95% CI: −0.51, 0.62] in HL, 0.63 [95% CI: −0.03, 1.24] in BFR_40_ and 0.07 [95% CI: −0.50, 0.63] in BFR_80_. At 24 h, effect size from baseline in LL was 0.13 [95% CI: −0.09, 0.70], in HL was −0.23 [95% CI: −0.80, 0.35], in BFR_40_ was 0.44 [95% CI: −0.17, 1.02] and in BFR_80_ was 0.07 [95% CI: −0.50, 0.63].

#### 3.2.6 SICI

**FIGURE 4:**
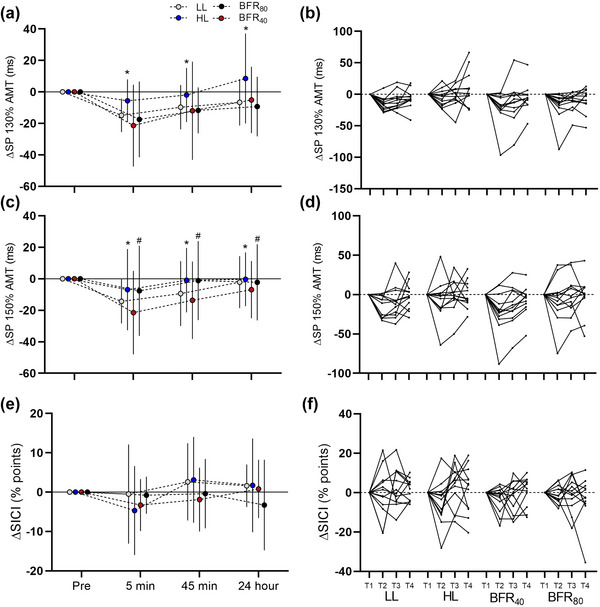
Change of inhibition measures derived from TMS over each time point in each experimental condition. (a) Mean ± SD of TMS silent period at 130% of active motor threshold. (b) Individual traces of SP130% AMT. (c) Mean ± SD TMS silent period at 150% of active motor threshold. (d) Individual traces of SP150%. (e) Mean ± SD short interval intracortical inhibition. (f) Individual responses of short interval intracortical inhibition. T1, T2, T3 and T4 denote pre, 5 min, 45 min and 24 h time points, respectively. *HL significantly greater than all other conditions (main effect of conditions *P *< 0.05). #BFR_40_ significantly lower than BFR_80_ (main effect of conditions *P *< 0.05).

There was no condition × time interaction for SICI (*F*_6,115.128_ = 0.835, *P* = 0.546). There was also no main effect of condition (*F*_3,88.177_ = 0.310, *P* = 0.818) or main effect of time (*F*_2,115.128_ = 1.861, *P* =  0.160) (Figure 4e).

### 3.3 Neuromuscular responses

**FIGURE 5:**
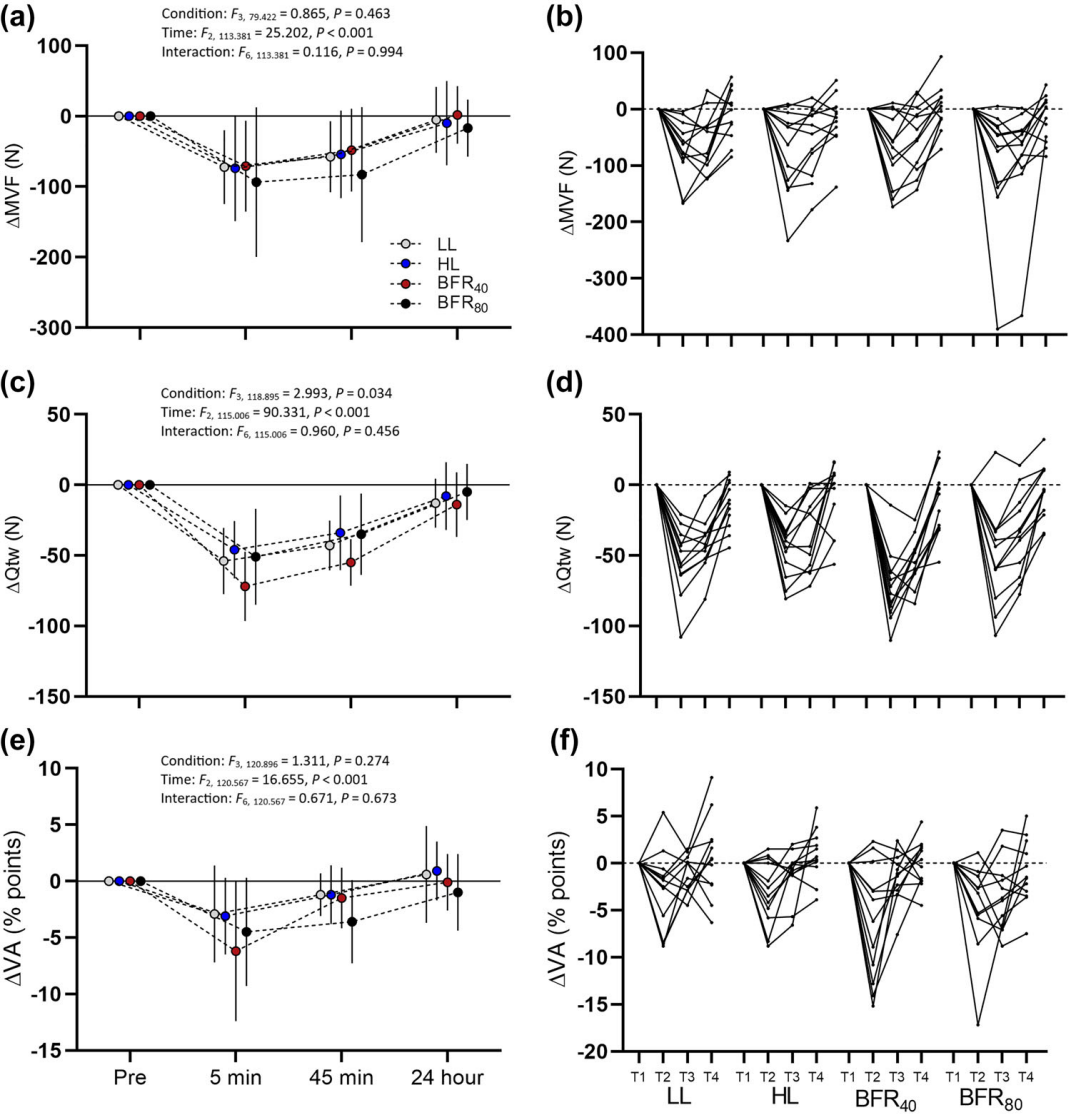
Changes in measures of neuromuscular function over time in response to each exercise protocol. (a) Mean ± SD of maximum voluntary force. (b) Individual traces of maximum voluntary force. (c) Mean ± SD quadriceps potentiated twitch force (peripheral fatigue). (d) Individual traces of quadriceps peripheral fatigue. (e) Mean ± SD voluntary activation (central fatigue). (f) Individual responses of voluntary activation.

Neuromuscular function changes and statistics can be seen in Figure 5. In short, there were no condition or interaction effects for MVF, VA or *M*_max_. A condition effect was observed for *Q*_tw_ (*F*_3,118.895_ = 2.993, *P* =  0.034) but post‐hoc tests failed to reveal significant differences (all *P *≥ 0.163, *d_z_* ≤ 0.65). Collapsed across conditions, the reduction in MVF from baseline indicated large effect sizes at 5 min of 1.31 [95% CI: 0.51, 2.01], 45 min of 1.26 [95% CI: 0.47, 2.01], with a small effect size at 24 h of 0.35 [95% CI: −0.24, 0.93]. For *Q*_tw_, there were also large effect sizes for reductions from baseline at 5 min of 3.06 [95% CI: 1.67, 4.43], 45 min of 2.72 [95% CI: 1.45, 3.96] and at 24 h of 0.88 [95% CI: 0.20, 1.54]. For VA, there were large effect sizes for reductions post‐exercise at 5 min of 1.23 [95% CI: 0.45, 1.97], 45 min of 1.23 [95% CI: 0.45, 1.97], but not at 24 h of −0.054 [95% CI: −0.62, 0.51].

### 3.4 Perceptual responses

There was no condition × set interaction for pain intensity (*F*_9,165_ = 1.737, *P* = 0.086). But there was an effect of set number (*F*_3,165_ = 40.728, *P* < 0.001) with pain intensity increasing linearly with each set. A condition effect was also observed (*F*_3,165_ = 96.366, *P* < 0.001) with pain intensity being greater in BFR_80_ compared to all other conditions (all *P *< 0.001, *d_z_* = 1.92–4.40), and with BFR_40_ being greater than HL and LL (both *P *< 0.001, *d_z_* = 1.95–2.48). Pain intensity was not different between HL and LL (*P *= 0.069, *d_z_* = 0.53). For RPE, a condition × set interaction was present (*F*_9,165_ = 2.806, *P* = 0.004). Relevant simple main effect comparisons can be observed in Table 3 with significant difference effect sizes ranging from *d_z_* = 0.68 to 2.49.

**TABLE 3:** Pain intensity and rating of perceived effort at the final rep of each set for each exercise protocol.

	Set 1	Set 2	Set 3	Set 4
Pain intensity (0–10)				
LL	3 ± 1	3 ± 1	4 ± 1	4 ± 2
HL*	2 ± 1	4 ± 1	5 ± 2	5 ± 2
BFR_40_*^†^	4 ± 2	5 ± 2	6 ± 2	7 ± 2
BFR_80_*^†‡^	5 ± 2	7 ± 2	9 ± 2	9 ± 1
Perceived effort (6–20)				
LL	13 ± 1	13 ± 2	13 ± 2	14 ± 2
HL	14 ± 1	15 ± 1*	16 ± 1*	17 ± 2*
BFR_40_	14 ± 2	15 ± 2*	16 ± 2*	17 ± 3*
BFR_80_	14 ± 2*	17 ± 2*^†‡^	18 ± 2*^†‡^	19 ± 1*^†‡^

*Note*: Symbols next to condition name represent significant difference as a main effect of condition. Symbols at individual data points represent differences within the interaction effect.

*Significantly different from LL (*P *< 0.05). †Significantly different from HL (*P *< 0.05). ‡Significantly different from BFR_40_ (*P *< 0.05).

### 3.5 Relationships

Exploratory analysis of relationships using a Hierarchical regression with ΔPPT from pre to 5 min in BFR_40_ as the dependent variable revealed no predictive capability of ΔMEP·*M*_max_, ΔSP130, ΔSICI, mean pain intensity during leg press exercise or ΔMVF (*F*_5,11_ = 0.344, *P* = 0.869). Similarly, as ΔSP130 revealed large effect sizes for changes from pre to 5 min, a separate linear regression model using ΔCSE130, ΔPPT, ΔSICI, mean pain intensity during leg press exercise or ΔMVF as predictors revealed no significant regression model on ΔSP130 (*F*_5,11_ = 0.071, *P* = 0.995).

## 4 DISCUSSION

The primary findings of the present study suggest that low‐load, BFR resistance exercise enhances hypoalgesia in comparison to low‐load, free flow exercise, but not high‐load, free flow exercise. Furthermore, BFR exercise at 80% LOP did not induce different hypoalgesic responses to BFR at 40% LOP despite greater perceptions of pain during the exercise. The increases in PPT does not appear to coincide with alterations of CSE or corticospinal inhibition. However, low‐load exercise appeared to reduce corticospinal inhibition (reflected with the TMS silent period) to a greater extent than high‐load exercise.

### 4.1 Exercise‐induced hypoalgesia

At the 5‐min post‐exercise time point there was a greater increase in PPT (1.54–1.94 kg force) of the exercised leg across HL, BFR_40_ and BFR_80_ in comparison with LL (Figure 2a). Importantly, the magnitude of these changes exceeded the upper confidence interval for the MDCs (>1.01 kg force; Table 1). However, at the 45‐min and 24‐h time points, PPTs had returned to baseline levels (Figure 2a). This return to baseline is consistent with previous literature that EIH typically only lasts 30–45 min (Naugle et al., 2012) and could be mediated by the transient production of endogenous opioids, which are not different from baseline at 24 h post‐exercise (Hughes & Patterson, 2020). We also failed to detect any systemic hypoalgesic response in non‐exercised body parts (contralateral quadricep, ipsilateral bicep and contralateral trapezius), which is in contrast to recent literature utilising BFR (Hughes & Patterson, 2020; Song et al., 2022, 2021b), but is in agreement with other studies which have employed a similar single‐blinded approach (Karanasios et al., 2022; Varangot‐Reille et al., 2022). The reason for the lack of systemic EIH is unclear (in comparison to previous findings), but in general may be due to the employment of a single, unilateral, dynamic resistance exercise protocol, which may not be a sufficient amount of exercising muscle mass and/or exercise volume to trigger systemic EIH in all participants.

The addition of high pressure BFR (80% LOP) to low‐load resistance exercise did not augment localised EIH to a greater extent than lower pressure (40% LOP) BFR, which is in contrast to Hughes and Patterson (2020), who found greater (and longer) increases in PPT with 80% LOP BFR exercise compared to free flow low‐load and high‐load exercise. This discrepancy in findings was unexpected because the same exercise and BFR interventions were performed; however, one explanation may be that the participants within this study experienced a greater degree of effort and pain (Table 3). As the degree of hypoalgesia may be mediated (amongst other factors) by the amount of exercise‐induced pain/discomfort (Bement et al., 2008; Hughes & Patterson, 2020, 2019) and the recruitment of high threshold motor units (Song et al., 2021a), it is possible that the hypoalgesic response was maximised in HL, BFR_40_ and BFR_80_ by performing a sufficiently effortful and painful exercise bout. To illustrate, peak pain ratings in our study were on average 1.5–2 points greater in HL, BFR_40_ and BFR_80_ (reaching 5–9 out of 10; strong to very strong pain) compared to the same protocols in Hughes and Patterson (2020), whereas peak effort rating were about 1 point greater, reaching 17–19 (very hard). Indeed, it has been observed in a subsequent investigation that when resistance exercise is performed to the point of momentary muscular failure (i.e., equally maximal effort levels and high; 6–7.5/10 pain), hypoalgesic responses are not different between free‐flow and BFR protocols (Song et al., 2022). Therefore, the addition of BFR does not appear to induce more EIH when exercise is sufficiently challenging, for example, peak pain of ‘strong pain’ 5/10 (Vaegter & Jones, 2020), RPE of ‘very hard’ 17/20 (Table 3). However, it should be considered that comparable hypoalgesic responses can be obtained with BFR to that of high‐load resistance exercise, although we acknowledge that there is limited generalisability in PPT changes in healthy individuals, as the EIH response may be altered in clinical populations (Rice et al., 2019).

### 4.2 Corticospinal inhibition responses

A novel finding of the present study was that the TMS silent period was lower in LL, BFR_40_ and BFR_80_ compared to HL (Figure 4a). Reflective of corticospinal inhibition mediated by GABA_b_ activity, the silent period appears to reduce after a bout of strength or motor skill training (Kidgell et al., 2017; Mason et al., 2019b). Acute changes to inhibitory neural circuits may be a key neurological adaptation to these types of training (Kidgell et al., 2017; Tallent et al., 2021) as well as a response to increased or decreased pain (de Almeida Azevedo et al., 2022; Norbury et al., 2022; Rio et al., 2015). However, we did not observe any relationship between the change of any TMS‐derived measurements of inhibition and the change in PPT, indicating that hypoalgesia did not coincide with a decrease in silent period duration or vice versa. Alternatively, the reduced inhibition within all low‐load conditions relative to the high‐load condition suggests that the number of externally paced repetitions modulated the inhibitory changes post‐exercise. Within HL, 40 repetitions were performed whereas in LL, BFR_40_ and BFR_80_, there was almost double the number of repetitions (∼75). It is plausible that the greater number of externally paced repetitions in the low‐load conditions acted as more potent stimulus for reducing corticospinal inhibition through changes in neural plasticity via motor skill training. Such findings are in contrast to previous work which has found that externally paced, high‐load strength training reduces TMS silent period to a greater extent than low‐load exercise (Mason et al., 2019a) whereas others have failed to detect a reduction in silent period (Colomer‐Poveda et al., 2020; Painter et al., 2020).

Short interval intracortical inhibition assessed with paired pulse TMS did not demonstrate any significant change across conditions or time. The amplitude of the conditioned MEP to the unconditioned MEP is thought to reflect GABA_a_ activity, which has previously been observed to decrease after strength training in some studies, but meta‐analysis suggests that this may not be the case (Mason et al., 2019b). Similarly, the addition of BFR does also not change this response after exercise (Brandner et al., 2015).

### 4.3 Corticospinal excitability responses

Unlike corticospinal inhibition, no change in MEP·*M*_max_ was observed between conditions for stimulation intensities at 130% and 150% of AMT (Figure 3a, c). To date, limited work has investigated the acute corticospinal responses to BFR exercise. One previous study (Brandner et al., 2015) observed greater increases in MEP·*M*_max_ for up to an hour after BFR was applied continuously (i.e., inflated during rest periods) during elbow flexion exercise in comparison to high‐load exercise. It is important to note that in that study the test muscle was the biceps brachii, which has greater corticospinal projections than the quadriceps (Brouwer & Ashby, 1990), which may partly explain the differences in findings. Additionally, the test contractions during the assessment of CSE in Brandner et al. (2015) were performed at the same absolute intensity (bodyweight supinated arm) despite potentially greater decreases in MVF induced by BFR exercise (Fatela et al., 2016; Hill et al., 2022; Husmann et al., 2018). As a result, the test contractions for CSE may have been performed at a greater relative intensity in the BFR conditions (compared to non‐BFR), and given that MEP amplitude increases as a function of the relative contraction strength (Gelli et al., 2007; Oya et al., 2008) up to about 50% of maximum force (Taylor et al., 1997), this may also partly explain the greater facilitation of MEPs with the BFR exercise. An interesting and novel finding within the present study was that AMT was lower in BFR_40_ compared to HL (Figure 3e), which is indicative of increased excitability of some lower‐threshold motoneurons (Hallett, 2007). This indicates that BFR at lower occlusion pressures may be superior to high‐load exercise at increasing excitability of the corticospinal pathway. However, the lack of difference between other conditions limits this interpretation. Furthermore, a parallel facilitation in MEP amplitudes would also be expected given an increase in excitability. A further consideration with these findings was that assessment of the corticospinal pathway was not segmented into spinal or supraspinal components. The measurement of lumbar evoked potentials induced by electrical stimulation of spinal pathways would have provided further insight into neurophysiological changes induced by BFR exercise (Gomez‐Guerrero et al., 2023). Therefore, future work should seek to investigate the cortical and spinal responses to BFR resistance exercise.

In relation to hypoalgesia, motor evoked potential amplitude is shown to positively correlate with more efficient conditioned pain modulation (Granovsky et al., 2019). Whilst not measured in this study, conditioned pain modulation is thought to share similarities with EIH (Vaegter et al., 2014) and potentially act as a mechanism (Ellingson et al., 2014), which is why an increase in MEP·*M*_max_ was expected. Differences in findings may be due to measuring CSE in a non‐motor versus motor (i.e., contracting muscle) state (Burns et al., 2016; Siddique et al., 2020). Alternatively, EIH and conditioned pain modulation may have distinct neurophysiological responses, but this requires further investigation.

Acute increases in CSE are also expected to occur after strength training (Tallent et al., 2021), but this has not consistently been observed (Kidgell et al., 2017; Tallent et al., 2021), with the findings of the present study supporting the notion that acute increases in CSE do not occur after strength training. However, more recent work by Alibazi et al. (2021) observed increases in knee extensor MEP amplitude with high‐load, but not low‐load knee extension exercise. Such findings are in contrast to the present study, which saw no increase in CSE in the HL condition despite externally pacing the exercise with a metronome, which is shown to promote increases in CSE (Ackerley et al., 2011; Leung et al., 2017) compared to self‐paced exercise. However, even metronome paced high‐load resistance exercise has failed to acutely increase CSE in the knee extensors (Ansdell et al., 2020). One reason for the discrepancy in CSE alterations may be due to the amount of neuromuscular fatigue induced by a resistance exercise protocol. Within the present study and in Ansdell et al. (2020), there was a prolonged (∼10%–20%) depression of MVC force post‐exercise, whereas in Alibazi et al. (2021), no reductions in knee extensor force were found after training. Given that fatiguing quadriceps exercise may reduce CSE (Goodall et al., 2018), there is likely a balance (or interaction) between use‐dependent plasticity (Tallent et al., 2021), neuromuscular fatigue (Goodall et al., 2018), and pain facilitation/inhibition (Granovsky et al., 2019; Sanderson et al., 2021) with the relative contribution of each factor determining the net CSE change.

### 4.4 Neuromuscular fatigue

As expected, the resistance exercise protocol revealed large estimated effect sizes for the presence of central and peripheral fatigue (Figure 5c,e), with central fatigue recovering completely within 24 h, but peripheral fatigue having not fully recovered, with large effect sizes indicating a reduction in *Q*_tw_ 24 h post‐exercise. Consequently, MVF was reduced (i.e., due to both central and peripheral factors) but had completely recovered by 24 h post‐exercise (Figure 5a). It is likely that there are immediate post‐exercise differences in the magnitude of neuromuscular fatigue between BFR and free flow exercise (Hill et al., 2022; Husmann et al., 2018), with a greater amount of central fatigue during BFR, mediated by the stimulation group III/IV afferents (de Almeida Azevedo et al., 2022; Hill et al., 2022; Norbury et al., 2022), and a greater magnitude of peripheral fatigue from reduced locomotor muscle oxygenation (Amann et al., 2006; Yanagisawa & Sanomura, 2017). Given that these factors recover substantially from the point of exercise termination (Husmann et al., 2018), this may explain why there were no differences observed in *Q*_tw_ or voluntary activation at the 5 and 45 min time points. Taken together, these findings suggest that BFR exercise does not induce a larger difference in the magnitude or time course (i.e., recovery) of neuromuscular fatigue than free‐flow or high‐load exercise in the acute (5 min to 24 h) post‐exercise.

### 4.5 Perceptual responses

Muscle pain displayed significant differences between conditions with a greater intensity of pain reported during the BFR_40_ and BFR_80_ conditions compared to both HL and LL. Furthermore, BFR_80_ was perceived to be more painful than BFR_40_ in the latter three sets, with pain reaching near‐maximal levels at the third and fourth set (Table 3), which is similar to Hughes and Patterson (2020), except values were greater in our study. Similarly, low‐load BFR resistance exercise has also been shown to induce more discomfort with higher compared to lower pressures when exercise is performed to momentary muscular failure (Dankel et al., 2019). Taken together, these findings indicate that BFR exercise is more painful to perform, particularly when higher occlusion pressures are used, and in comparison to free‐flow exercise (Spitz et al., 2022). This is due to the greater accumulation of noxious biochemicals (Pollak et al., 2014) induced by limited venous outflow of the working musculature. Future research should quantify changes in the quality of pain and the unpleasantness (i.e., measure affect) with BFR to gain a greater insight into perceptual responses.

Similar to pain intensity, perceptions of effort were greater in BFR_40_ and BFR_80_ compared to HL and were greater in BFR_80_ compared to BFR_40_ (Table 3). The definition of effort employed in this study (and instructed to participants) was the ‘effort to drive the limb’ and did not factor perceptions of pain, discomfort or fatigue. The perception of effort is thought to be caused by an efferent copy of the corollary discharges which are processed in sensory areas of the brain (de Morree et al., 2012) and not directly from the afferent feedback during exercise (Bergevin et al., 2022). Given that BFR exercise is more fatiguing during the exercise task than its free‐flow counterpart (Husmann et al., 2018), it is likely that the RPE reflected an increased central motor command due to the recruitment of high threshold motor units to compensate for the accelerated neuromuscular fatigue with BFR (de Morree et al., 2012; Fatela et al., 2019).

### 4.6 Methodological considerations

Some participants were unable to complete all of the prescribed repetitions in the exercise protocols (see Table 2). In particular, during BFR_80_ there were five participants who were unable to complete more than 90% of the total repetitions. The lower exercise volume may have impacted some of the neuromuscular fatigue and neurophysiological responses, but the mean completion rate was 88% (66/75 repetitions) and so we contend the impact of this would have been minor. Nevertheless, we included exercise volume as a covariate in our statistical models, and in most cases, exercise volume did not significantly influence the dependent variable of interest. This does, however, highlight the challenge that even when restriction pressures and exercise intensities are set relative to the individual, there is still a significant heterogeneity in completion ability. Therefore, future work may want to either standardise protocols to induce momentary muscular failure or implement a fixed effort/pain protocol (e.g., repetitions in reserve) to further study the effects of BFR exercise.

The inclusion of a control condition (i.e., time‐matched quiet rest) would have allowed for stronger inferences to be made about changes over time. However, comparisons between exercise protocols were of primary interest, and comparing BFR exercise at low and high pressures to free‐flow low and high‐load exercise provides a more comprehensive insight about the effects of BFR on neurophysiological and hypoalgesic parameters.

Finally, this study was limited to healthy, pain free males. Therefore, these findings will have limited application to female populations, as it has been demonstrated that females demonstrate different profiles and recovery of neuromuscular fatigue (Ansdell et al., 2019). Furthermore, psychophysiological responses (e.g., pain and effort) to BFR exercise have been suggested to be exacerbated in females compared to males (McClean et al., 2023) and these findings may translate differently to those who experience pain in the rehabilitative period (e.g., after anterior cruciate ligament reconstruction) or those who suffer from chronic pain conditions.

### 4.7 Conclusion

In summary, low‐load BFR resistance exercise induces local EIH for a short period of time in comparison to free‐flow, low‐load exercise but does not surpass free‐flow, high‐load exercise. Excitability or inhibition of the corticospinal pathway was not associated with an increase in hypoalgesia as hypothesised, but low‐load exercise reduced corticospinal inhibition to a greater degree than high‐load exercise. Therefore, the central mechanisms of EIH induced by BFR are likely not mediated by GABA responses and require further investigation. Individuals who implement BFR resistance exercise for training and rehabilitation purposes can do so without inducing different neurophysiological responses or causing a greater acute level of neuromuscular fatigue. However, the addition of BFR will be more painful and effortful compared to high‐load exercise or free‐flow low‐load exercise, and this should be taken into consideration when prescribing BFR exercise, particularly for individuals who are not accustomed to painful exercise.

## AUTHOR CONTRIBUTIONS

All experiments were performed in the laboratory facilities at St Mary's University, Twickenham. Ryan Norbury, Luke Hughes, Alex Woodhead, Jamie Tallent and Stephen Patterson contributed to the conception and design of the study. Ryan Norbury, Ian Grant and Alex Woodhead were responsible for acquisition, analysis, or interpretation of data for the work. Ryan Norbury, Ian Grant, Luke Hughes, Alex Woodhead, Jamie Tallent and Stephen Patterson drafted the work and revised it critically for important intellectual content. All authors have approved the final version of the manuscript, and all authors agree to be accountable for all aspects of the work in ensuring that questions related to the accuracy or integrity of any part of the work are appropriately investigated and resolved. All persons designated as authors qualify for authorship, and all those who qualify for authorship are listed.

## CONFLICT OF INTEREST

All authors declare no competing interests with this research.

## Supporting information

Supporting Information

## Data Availability

The raw data for the study is available online at: https://doi.org/10.6084/m9.figshare.24156741. Availability of specific analysis files can be provided upon reasonable request.
